# An assessment of video-observed treatment as an adherence support tool for patients with drug-resistant TB

**DOI:** 10.5588/ijtldopen.25.0370

**Published:** 2025-12-10

**Authors:** B.B. Nkala, A. Kay, A. Vasiliu, A. Mandalakas, T. Nkomo, L. Mdluli-Dlamini, S. Ngwenya, S.S. Thi, S. Masina, J. Sibanda, S. Tsela, D. Vambe

**Affiliations:** 1 Baylor College of Medicine Children’s Foundation Eswatini, Mbabane, Eswatini;; 2 Department of Pediatrics, Baylor College of Medicine, Global TB Program, Houston, TX, USA;; 3 Research Center Borstel, Division of Clinical Infectious Diseases, Borstel, Germany;; 4 German Center for Infection Research (DZIF), Partner Site Hamburg-Lübeck-Borstel-Riems, Hamburg, Germany;; 5 Eswatini National Tuberculosis Control Program, Manzini, Eswatini.

**Keywords:** tuberculosis, mobile health technology, patient-centred care, digital adherence technology

## Abstract

**BACKGROUND:**

In 2017, WHO endorsed digital adherence technologies, including video-observed treatment (VOT), to support TB treatment adherence. In 2021, Eswatini implemented VOT to support adherence for drug-resistant TB (DR-TB) patients nationwide, although limited evidence exists regarding its feasibility and acceptability in low-resource settings.

**METHODS:**

We conducted 33 in-depth interviews with DR-TB patients and health care providers (HCPs), across all four regions of Eswatini to explore their experiences with VOT.

**RESULTS:**

The findings indicated broad acceptance of VOT among both HCPs and patients. HCPs reported that VOT strengthened their connection with patients, facilitated adherence, and increased their confidence in treatment outcomes. While most patients found VOT user-friendly and appreciated the independence and privacy it offered, elderly patients faced challenges using smartphones, which sometimes resulted in poor-quality video submissions.

**CONCLUSION:**

VOT is feasible and acceptable among HCPs and patients in Eswatini. Further research is needed to evaluate the impact of VOT on treatment outcomes in high-burden and low-resource settings to inform the scale-up of similar digital health interventions in comparable contexts. Strengthening digital infrastructure and providing tailored support for elderly patients could further enhance VOT effectiveness and equity.

TB is one of the top 10 causes of deaths globally, with about 10.8 million new cases reported in 2023, of which 75% were notified.^1^ In Eswatini, 65% of TB patients are co-infected with HIV, with an even higher co-infection rate of 71% among patients with drug-resistant TB (DR-TB).^1^ Adherence to DR-TB treatment is challenging due to the complex management of DR-TB and HIV medications, as well as potential drug-to-drug interactions.^2^ To address these challenges within budget constraints, Eswatini introduced video-observed treatment (VOT) specifically aiming to enhance adherence monitoring and facilitate timely reporting of adverse events.^3^ Adherence to TB treatment is critical for successful outcomes but remains a significant challenge among patients with DR-TB. In-person treatment support (pTS) or directly observed treatment (DOT) is globally recommended for patients with DR-TB; however, it can be disempowering and stigmatising for patients.^4^ While pTS may accomplish the goal of ensuring adherence to medication, it does so at a cost to both the patient and to the health system.^5^ Furthermore, when implemented in low-resource, high-burden settings, pTS responsibility often falls to community and family members, risking the spread of TB infection, stigmatisation of patients, and compromising the goal of ensuring good adherence to treatment.^6–8^

To address these challenges, VOT has been proposed as an innovative solution to improve patients’ adherence to DR-TB treatment.^9–12^ VOT allows health care providers (HCPs) to remotely support patients’ adherence, address adverse events, and reduce the need for in-person clinic visits.^8,10,13^ Previous studies have shown that VOT can increase adherence rates and lessen the burden of in-person visits for patients.^10,12,14,15^ However, little is known about its feasibility in low- and middle-income countries (LMICs), where technological limitations such as internet access and digital literacy may present barriers.^16^ Furthermore, VOT feasibility for health systems has been primarily studied in locations where the public health system is responsible for treatment monitoring which is not typically the case in LMICs settings, where treatment support often is deferred to family and community members.^17,18^ VOT allows patients to take medication at their convenient time and receive adherence support from HCPs via a smartphone; however, the feasibility and acceptability of VOT implementation in low-resource, high-burden settings is limited.^13,19^

This study aimed to assess both patients’ and HCPs’ perceptions on the feasibility and acceptability of VOT from a nationally representative sample.

## METHODS

In this qualitative study, we conducted in-depth interviews using interview guides. The study included two key participant groups represented by patients with DR-TB who were enrolled in the VOT programme and HCPs involved in supporting patients on VOT. To ensure a diverse population of patients, seven DR-TB health facilities located in all four regions of the country were purposively selected. Patients aged 18 years and older who had used VOT for at least 3 months were selected from Baylor COE TB Clinic, Manzini TB Center, Dvokolwako Health Center, Hlathikhulu Government Hospital, Nhlangano Health Center, Good Shepherd Hospital, and AIDS Health Foundation (AHF) Matsapha Clinic. Participants were invited to participate in the study through their respective health facilities during follow-up or through a scheduled visit, and informed consent was obtained prior to participation. HCPs included registered nurses, nursing assistants, and adherence officers who interacted directly with the VOT system.

### Interview conduct

In-depth semi-structured interviews were conducted with patients and HCPs using their preferred language. A total of 33 interviews were conducted lasting between 20 and 45 min per interview to allow for an in-depth exploration of participants’ experiences. Interviews were conducted in a private and comfortable setting to ensure participants felt at ease and could speak candidly. Interviews conducted in Siswati were transcribed and translated into English. Anonymised transcripts were reviewed for accuracy, and errors or omissions were corrected before the analysis.

The interviews aimed to explore participants’ personal experiences with VOT, including their challenges, benefits, barriers, facilitators, and overall satisfaction. We aimed to interview all the participants who had undergone VOT during their treatment, and data saturation was achieved following 33 interviews.

### Analysis

A thematic analysis was used to identify and analyse patterns and themes. This involved coding the data into categories, grouping similar codes into themes, and interpreting these themes in relation to the research questions. Constant comparison was made to refine themes and ensure they accurately reflect participants’ perspectives. NVivo version 14.23.3 (61) was used to analyse the data. The findings were validated through a review process, where a reviewer was asked to examine and confirm the accuracy of the themes and interpretations derived from the interviews.

### Ethical statement

Ethics approvals were obtained from the Baylor College of Medicine Children’s Foundation Eswatini Institutional Review Board and the Eswatini Human Health Research Review Board (EHHRRB). All participants provided written informed consent prior to participation.

## RESULTS

A total of 11 HCPs were interviewed (Table 1). There were 6 nurses (54.5% of the group) who, together, contributed 12 years of VOT experience, averaging 2 years per nurse. In addition, 3 nursing assistants (27.3%) collectively had 6 years of experience, while two adherence officers (18.2%) contributed a total of 5 years of experience.

**Table 1.** Characteristics of study participants.

DR-TB clients (n = 22)			
Characteristic	Category	Frequency (n)	Percentage (%)
Sex	Male	12	55%
	Female	10	45%
Age group (years)	18–30	3	13%
	31–45	10	46%
	46–60	5	23%
	>60	4	18%
Region	Hhohho	6	27%
	Manzini	5	23%
	Shiselweni	8	36%
	Lubombo	3	14%
Facility location	Urban	4	57%
	Rural	3	43%
Duration on VOT	3–6 months	20	91%
	>6 months	2	9%
HCPs			
Professional cadre	Number of HCPs	Percentage	Average years per HCP
Registered nurses	6	54.5%	2.0
Nursing assistants	3	27.3%	2.0
Adherence officers	2	18.2%	2.5
Total	11	100%	—

VOT = video-observed treatment; DR-TB = drug-resistant TB; HCPs = health care providers.

### Perceived benefits of VOT by HCPs

Most HCPs (91%) reported that VOT significantly improved treatment adherence among patients and 73% increased patient independence (Table 2). Other benefits such as reduced stigma (64%) and enhanced confidentiality (55%) were also widely observed. Most HCPs (82%) expressed a clear preference for VOT over DOT, while only one HCP preferred the traditional DOT method. The reasons for preferring VOT included greater convenience for patients, increased patient autonomy, and improved adherence. Improved adherence (91%) and increased patient independence (73%) were the most frequently cited facilitators of VOT. The most frequently cited challenge was network and data issues, which 82% of HCPs observed. More than half (55%) of HCPs cited patients’ technological literacy as a barrier among elderly patients. A considerable proportion of HCPs (45%) reported that VOT increased their workload due to the time required to view the videos. However, 36% of HCPs indicated that their workload had decreased as VOT reduced the need for frequent in-person observations. The remaining 18% reported no significant change in their workload.

**Table 2.** Perceived benefits of VOT by HCPs.

Category	Subcategory	Percentage (%)
Perceived benefits of VOT by HCPs	Improved treatment adherence	91%
	Increased patient independence	73%
	Reduced stigma	64%
	Enhanced confidentiality	55%
	Reduced health care workload	45%
Challenges of VOT implementation	Network and data issues	82%
	Technological literacy of patients	55%
	Time-consuming for HCPs	45%
	Insufficient patient training	36%
Effectiveness of VOT compared to DOT	VOT preferred	82%
	DOT preferred	9%
	Neutral (no strong preference)	9%
Impact on HCPs	Increased workload	45%
	Decreased workload	36%
	No significant change	18%
Challenges with technological literacy	High technological literacy (easy adaptation)	27%
	Moderate technological literacy	45%
	Low technological literacy (difficult adaptation)	27%
Recommendations for improving VOT	Ensure consistent data supply	73%
	Provide technological training	55%
	Offer financial incentives to patients	45%
	Reduce health care worker workload	27%

VOT = video-observed treatment; DOT = directly observed treatment; HCPs = health care providers.

A summary of barriers and facilitators is provided in Table 3 and paired with illustrative quotes in Table 4. To improve the usage of VOT, 73% of HCPs recommended ensuring a consistent mobile data provision to prevent video upload challenges. Additionally, 55% of HCPs suggested providing more comprehensive smart phone technology training on the use of smart phones for video-recording and uploading to the digital adherence information platform for patients, and 45% recommended offering incentives to motivate patients to adhere to treatment.

**Table 3.** Summary of perceived barriers and facilitators of VOT by HCPs.

Barrier/facilitator	Frequency (%)
Improved adherence (facilitator)	91%
Network/data issues (barrier)	82%
Increased patient independence (facilitator)	73%
Patient stigma reduction (facilitator)	64%
Technological literacy issues (barrier)	55%

VOT = video-observed treatment; HCPs = health care providers.

**Table 4.** Illustrative quotes for HCPs.

Theme	Quote
Benefits of VOT for HCPs	Increased treatment adherence: ‘The biggest benefit to me is that now I know almost 100% that the patient is taking the medication’ echoed this, emphasising that they could identify any issues with adherence in real-time, allowing for immediate intervention.
	Patient empowerment and self-reliance: ‘It has made privacy easy among patients. They take their medications privately without any stigma’.
	Reduction in stigma and increased confidentiality: ‘It helps them because they can take their medications privately, without the judgment of others in the community’.
Impact on HCP–patient relationship	Building trust and better communication: ‘Patients feel that we are concerned about their health because they know we are watching them daily’.
	Limited interaction for addressing side effects: ‘Patients often report side effects during their monthly visits rather than through the VOT platform’.
Recommendations for improving VOT	Consistent data supply: ‘There should be consistent data supply so that patients can upload their videos daily without interruptions’.
	Incentivising patients: ‘We could motivate patients more by offering them incentives for adhering to VOT, such as allowing them to keep the phone after treatment’.
Challenges encountered with VOT	Technical difficulties (network and data challenges): ‘There are areas where network coverage is poor, and the videos are not uploaded in time’.
	Technological literacy: ‘The elderly sometimes need assistance with the phone, and they can struggle to take a good quality video properly’.
Workload for HCPs	Workload for health care providers: ‘It’s time-consuming when there are many videos to watch, and we’re short-staffed’.
Future considerations for VOT	Expansion to other chronic conditions: ‘I would recommend VOT for managing chronic conditions because it helps patients adhere to long-term treatments’.

VOT = video-observed treatment; HCPs = health care providers.

### Characteristics of DR-TB patients

A total of 22 patients were interviewed, of which 12 (55%) were males and 10 (45%) were females (Table 1). The majority of the patients (82%) were between 18 and 60 years of age while those above 60 years accounted for 4 (18%).

### Perceived benefits of VOT among DR-TB patients

All participants received initial training, with 90% finding it useful and easy to use (Table 5). A significant majority (85%) felt that VOT enhanced privacy and confidentiality compared to the DOT adherence support method. The VOT system increased feelings of independence and ownership over health for 85% of respondents, and 75% reported enhanced connection through continuous HCP support. The VOT system was credited with improving adherence to medication schedules by 95% of participants, and 80% found alarm reminders helpful. A substantial proportion (70%) faced challenges with mobile network and irregular or delayed data availability. Participants provided feedback for improvement, with 40% suggesting a reduction in steps for video uploads and 25% recommending better video quality. Approximately 30% expressed a desire to retain the VOT mobile phone devices post-treatment.

**Table 5.** Summary of key findings from patients.

Theme	Response/observation	Percentage
Training and ease of use	Received initial training.	100%
	Found the training useful.	90%
	Reported that training was minimal or one-time only.	50%
	Found VOT app easy to use.	90%
	Suggested need for more training or follow-ups.	40%
Privacy and confidentiality	VOT improved privacy and confidentiality compared to having a treatment supporter.	85%
	Expressed concerns about potential data privacy issues (e.g., videos being shared without consent).	10%
Impact on adherence	VOT helped improve adherence to medication schedules.	95%
	VOT alarm reminders were found helpful.	80%
	Experienced occasional interruptions in adherence due to technical or network issues.	20%
Technological and network issues	Faced network and data issues (e.g., poor connectivity and depleted data).	70%
	Suggested improvement in data availability (e.g., providing additional data or offline functionality).	65%
Psychological and social impact	VOT increased independence and ownership of health.	85%
	Reduced emotional burden by having continuous monitoring by HCPs.	75%
Application functionality	Suggested VOT app improvement to reduce steps needed for video uploads.	40%
	Recommended better video quality and clearer video submission.	25%
Provision of devices	Expressed a desire to keep the VOT devices after treatment.	30%

VOT = video-observed treatment; HCPs = health care providers.

## DISCUSSION

In-depth interviews with HCPs and patients about the usage of VOT provided valuable insights into the feasibility, challenges, and potential areas for improvement. VOT was widely regarded as an acceptable tool for improving treatment adherence. Approximately 91% of HCPs reported higher adherence rates compared to DOT model, due to VOT’s ability to remotely support patients’ adherence. This aligns with other findings that VOT is a viable alternative for adherence support and reduces the need for in-person supervision and provides flexibility.^14,20–22^ A key benefit of VOT was the increased privacy and autonomy it provided to patients, empowering them to take responsibility for their treatment. This independence, especially in stigmatised communities, was deemed to be crucial in sustaining long-term adherence by both patients and HCPs and has been reported consistently across a variety of settings.^10–12^

However, despite these benefits, several challenges were identified. The most significant challenge was network connectivity and data-related problems, particularly in rural areas where network access is limited. Around 82% of HCPs noted that patients experienced difficulties with video uploads due to irregular or delayed data provision and poor network coverage. This has been observed as a consistent challenge with VOT in low-resource settings.^23,24^ The results also indicated that older patients with less mobile health literacy have many concerns engaging with telehealth and most need support to use telehealth, echoing findings from other studies.^25,26^ Enhancing the VOT platform with more pictorial guidance or incorporating infographics could make it more user-friendly for elderly patients and those with limited technological literacy. Visual cues can simplify navigation, making it easier for patients to understand recording steps and submission status, and reduce potential errors. Furthermore, reviewing video submissions proved to be time-consuming for HCPs, with about 45% stating that managing large volumes of video reviews increased their workload, despite VOT reducing the need for in-person visits. Digital approaches to primary care can increase general practice workload.^27^ Task shifting this activity to adherence officers or lay counsellors with oversight from nurses can reduce nurses’ work overload.

VOT positively impacted HCP–patient relationships. Despite its remote nature, HCPs felt that VOT facilitated closer communication and timely intervention as evidenced by other studies.^10,15,28^ While some of the benefits of VOT in Eswatini were also reported in high-income countries, the context of implementation differs significantly. In Eswatini, challenges such as intermittent mobile data, limited smartphone access, and varying levels of digital literacy were major barriers to seamless VOT adoption. These infrastructure and technological constraints contrast with VOT implementation in high-income settings, where patients typically have reliable internet, high device ownership, and greater familiarity with digital tools.^29,30^ Additionally, cultural norms around privacy, stigma, and health system capacity differ. For instance in Eswatini, family or community members often serve as treatment supporters, thus potentially increasing stigma, whereas in high-income countries adherence monitoring is usually handled by professional health care staff. These contextual differences underscore the need for tailored VOT strategies that account for local infrastructure, workforce capacity, and socio-cultural dynamics.^14,31^

### Recommendations for improving VOT

Participants provided key recommendations for improving the VOT system in Eswatini. The most suggested improvement by HCPs was ensuring timely and consistent mobile data supply to enable real-time monitoring of patient adherence. Additional training and support for patients, especially those unfamiliar with smartphones, was also recommended to address technological literacy challenges. Enhancing the uptake of VOT through task shifting to other cadres, such as adherence officers, may help reduce nurses’ workload while improving patient support and treatment adherence. Some HCPs suggested offering incentives similar to the traditional DOT model and giving the phone to patients who completed treatment as a gift, to encourage adherence. Patients on DOT typically receive E300 (US$17.64) for travel costs, while the treatment supporter, often a family member, receives E500 (US$29.41) per month, whereas patients on VOT received E300 only. Incentivising smartphones post-treatment was highly recommended by HCPs in lieu of the treatment supporter stipend.

Limitations of the study include the observation that some participants perceived the study as intrusive and were reluctant to provide honest feedback, resulting in social desirability bias, which may have influenced participants’ responses during interviews, particularly when discussing challenges of VOT. While data saturation was achieved, this may limit the diversity of perspectives and the depth of insights. Participants may have struggled to recall their experiences accurately, especially those involving technical issues or adherence challenges, leading to recall bias.

## CONCLUSION

While VOT offers a promising solution for improving DR-TB treatment adherence, this study highlights the need for context-specific adaptations in resource-limited settings like Eswatini. VOT has shown significant promise in improving adherence and reducing stigma, and addressing barriers such as data bundle depletion, lack of technological literacy, and HCPs’ workload will be critical to its success in resource-limited settings. Addressing these challenges is crucial for the success of VOT implementation. Expanding VOT to other chronic conditions, such as HIV and non-communicable diseases, could enhance adherence across diverse chronic conditions.
